# The Action of Cannabidiol on Doxycycline Cytotoxicity in Human Cells—In Vitro Study

**DOI:** 10.3390/molecules30214319

**Published:** 2025-11-06

**Authors:** Lidia Radko, Tatiana Wojciechowicz, Oliwia Kończak, Paula Żakowicz, Oskar Łętowski, Julia Salmanowicz, Zuzanna Skrzypczak

**Affiliations:** 1 Department of Preclinical Sciences and Infectious Diseases, Faculty of Veterinary Medicine and Animal Sciences, Poznan University of Life Sciences, 60-637 Poznan, Poland; 2 Department of Animal Physiology, Biochemistry and Biostructure, Faculty of Veterinary Medicine and Animal Sciences, Poznan University of Life Sciences, 60-637 Poznan, Poland; tatiana.wojciechowicz@up.poznan.pl; 3 Students Scientific Society of Veterinary Medicine, Section of Veterinary Pharmacology and Toxicology “Paracelsus”, Faculty of Veterinary Medicine and Animal Sciences, Poznan University of Life Sciences, 60-637 Poznan, Poland; 70907@student.puls.edu.pl (O.K.); paulazakowicz@gmail.com (P.Ż.); oskar.letowsky@gmail.com (O.Ł.); julia.salmanowicz2508@gmail.com (J.S.); zskrzypczak123@gmail.com (Z.S.)

**Keywords:** cannabidiol, doxycycline, interaction, human

## Abstract

Improper use of drugs in both animal and human therapy, such as doxycycline (DOX), lead to the accumulation of residues in edible animal tissues as well as in the environment. Plant-derived compounds reduce the adverse effects of drugs. This study aimed to evaluate the effect of cannabidiol (CBD) in two concentrations: lower (1.56 µg/mL) (DOX + C1) and higher (3.125 µg/mL) (DOX + C2) on the cytotoxicity of doxycycline in human cells. The toxicity of DOX and its CBD-containing mixtures was assessed after 72 h of exposure in three human cell lines: neural (SH-SY5Y), hepatic (HepG2), and kidney (HEK-293). The exposure to DOX resulted in inhibition of mitochondrial activity (SH-SY5Y) and inhibition of DNA synthesis (HepG2 and HEK-293). IC_50_ values for DOX ranged from 9.8 to >200 µg/mL in SH-SY5Y cells, 13.4 to 200 µg/mL in HepG2 cells, and 8.9 to 30.4 µg/mL in HEK-293 cells. The nature of the interaction depended on both the cell lines and the concentration of CBD in the mixture. Both CBD mixtures demonstrated a synergistic interaction in neuronal cells. In HepG2 cells, both mixtures showed additive and antagonistic interactions. In HEK-293 cells, the DOX + C1 mixture exhibited an antagonistic (protective) effect, while the DOX + C2 mixture showed an additive effect. There were no changes in oxidative stress levels; however, alterations in apoptosis levels and cell morphology were observed following exposure to the mixtures. The presence of doxycycline in the diet and the environment poses a health risk to consumers. The increasing consumption of CBD-containing products may reduce the risk associated with the presence of this drug in food. It is worth emphasizing the need for research aimed at minimizing the adverse effects of pharmaceuticals on the health of humans and animals.

## 1. Introduction

Amid growing concerns about food safety, increasing efforts are being made to develop dietary, technological, and regulatory strategies aimed at reducing the presence of harmful contaminants in food and minimizing the risk of their toxic effects on consumer health. Undesirable substances detected in animal-derived products—such as meat, eggs, and milk—as well as in the environment (including water, soil, and plants), include antibiotics, which are extensively used to treat both animals and humans.

One of the commonly used antimicrobial agents is doxycycline (DOX), a second-generation tetracycline antibiotic [1,2]. The antibiotic binds to the 30S ribosomal subunit, inhibiting bacterial protein biosynthesis [3]. Doxycycline is active against G+ and G− bacteria, atypical pathogens, and certain protozoa (e.g., *Plasmodium* spp.) [4]. Compared to other tetracyclines, doxycycline is more lipophilic, which allows for better tissue and fluid penetration. Due to its favorable pharmacological properties, DOX is extensively used in human and veterinary medicine, particularly in poultry and swine husbandry. Doxycycline-induced toxicity reports have been limited to accidental calf poisoning and rat experimentation poisoning [5,6,7,8,9]. However, a recent experimental trial in calves and rats failed to produce the cardiac lesions that had been reported in field cases. However, long-term therapy can result in its prolonged persistence in the body, leading to unacceptable residue levels in animal tissues. The widespread use of antibiotics contributes to contamination of both the environment [2] and the food chain [10,11,12,13,14]. According to a report by the European Medicines Agency (EMA), tetracycline antibiotics ranked second among the most frequently sold antimicrobial agents for food-producing animals, accounting for 21.6% of total sales [14]. As a result, tetracyclines are among the most commonly detected veterinary drugs in food monitoring studies.

In accordance with European Union Regulation No. 37/2010, maximum residue limits (MRLs) for doxycycline in edible tissues have been established: 600 μg/kg for kidneys, 300 μg/kg for liver, and 100 μg/kg for muscle. No MRL has been set for DOX in eggs or milk, meaning that the use of this antibiotic is prohibited in laying hens and dairy cows [13]. Nevertheless, residues of DOX have been detected at levels up to 1400 μg/kg in eggs and 5–490 μg/kg in milk [15,16]. Studies have shown that DOX accumulates at the highest concentrations in the kidneys (44.7 ± 4.3 μg/kg). The concentrations measured in the liver were slightly lower (21.6 ± 3.2 μg/kg). In breast and thigh muscles, the levels of the drug were 32.4 ± 3.6 μg/kg and 22.7 ± 2.9 μg/kg, respectively [15,17]. Additionally, it should be emphasized that heat treatment does not eliminate DOX residues from tissues [18,19]. The presence of doxycycline has been reported not only in food of animal origin [20] but also in vegetables [21], and even in drinking water [22].

It has been found that contamination of food with antibiotics can have various adverse effects on human health, even at low concentrations and with long-term exposure [23,24,25]. Exposure to doxycycline has been associated with the development of obesity and type 2 diabetes [3]. This antibiotic disrupts the human gut microbiota [26,27,28]. Even low concentrations of the antibiotic can contribute to multidrug resistance [29] and increase the prevalence of antibiotic resistance genes [30]. Notably, even short-term administration of the antibiotic can enable resistant bacterial populations to stabilize and persist in the human body for years [28].

The danger associated with exposure to doxycycline (DOX) also lies in its toxic effects on the gastrointestinal tract, liver, kidneys, skin, bones, and nervous system [24,25]. Doxycycline exhibits hepatotoxic properties [4]. Among the adverse effects are photosensitivity, skin lesions, and itching. DOX is particularly hazardous to young, developing individuals due to its accumulation in teeth and bones, which can lead to tooth discoloration, impaired bone growth, and skeletal deformities [4]. Prolonged exposure may also lead to neurological symptoms such as headaches and dizziness [31,32]. It has been shown that this antibiotic can enhance the neurotoxic effects of other concurrently administered drugs. The risk of neurotoxicity may vary depending on individual factors such as age, kidney function, and pre-existing neurological conditions [32]. Special attention should be paid to interactions between doxycycline and other medications. It is well known that antacids containing magnesium, calcium, aluminum, or iron salts can impair its absorption. Cytochrome P450 3A4 inducers (such as rifampicin) reduce plasma concentrations of doxycycline due to increased hepatic metabolism. Anticonvulsants like phenytoin and carbamazepine, as well as barbiturates, acetazolamide, and sodium bicarbonate, also lower serum levels of the drug. Additionally, taking doxycycline may reduce the effectiveness of oral contraceptives. It may also interfere with cancer therapies by interacting with cytotoxic drugs such as methotrexate. Doxycycline has been shown to displace methotrexate from its binding sites, leading to elevated methotrexate levels and increased toxicity [4].

An important public health protection strategy is to minimize the adverse effects of antibiotic residues in food and the environment through the use of natural active substances. The growing interest in and sales of hemp-derived products containing cannabidiol (CBD) oils may offer a potential solution to this problem. CBD is a naturally occurring cannabinoid found in *Cannabis sativa*. Importantly, it does not exhibit psychoactive effects and has no addictive potential, which contributes to its favorable safety profile. Studies have demonstrated that CBD exerts multifaceted cytoprotective effects in models of toxic damage to the kidneys [33,34], liver [35,36], and nervous system [37,38].

Three human cell lines were used in this study as models of neuronal cells (SH-SY5Y line), liver cells (HepG2 line), and kidney cells (HEK-293 line). The SH-SY5Y neuronal cell line is a widely used human neuroblastoma cell line. These cells are frequently employed in scientific research to analyze various aspects of neurobiology [39]. They are also used in the screening of potentially neurotoxic compounds and in evaluating drug candidates for disorders of the central nervous system [40]. The HepG2 cell line is derived from a human liver tumor (hepatoma) and exhibits many functional characteristics typical of liver cells, making it a valuable tool in toxicity and metabolism studies [41,42]. It is one of the most commonly used models in cytotoxicity testing, particularly in the context of assessing chemicals, drugs, and other compounds that may affect the liver. Although SH-SY5Y and HepG2 cell lines are widely used in many studies, they also have certain limitations—primarily their cancerous origin, which means they do not fully replicate all aspects of normal neuronal function or the complex in vivo environment. Nevertheless, in in vitro research, they serve as valuable cell models that allow for the determination of concentration ranges and mechanisms of action of specific substances.

This innovative study evaluated the effects of cannabidiol (*Cannabis sativa* L.) on doxycycline-induced toxicity using three cell cultures: neural—SH-SY5Y, liver—HepG2, and kidney—HEK-293. The cultured cells, for 72 h, were exposed to doxycycline (at concentrations ranging from 1.56 μg/mL to 200 μg/mL), both alone and in combination with CBD: 1.56 μg/mL (DOX + C1) and 3.125 μg/mL (DOX + C2) at two non-toxic doses (Supplementary Materials). A series of cytotoxicity assays included assessments of mitochondrial activity, lysosomal activity, cell membrane integrity, DNA synthesis, and cell proliferation. Based on the obtained results, IC_50_ values for doxycycline, both in monotherapy and in combination with CBD, were calculated, along with evaluations of ROS, death of cells, and morphology. The interaction between the drug and CBD was further analyzed by the combination index (CI).

## 2. Results

### 2.1. The Effects of CBD on Doxycycline’s Activity

**Figure 1:**
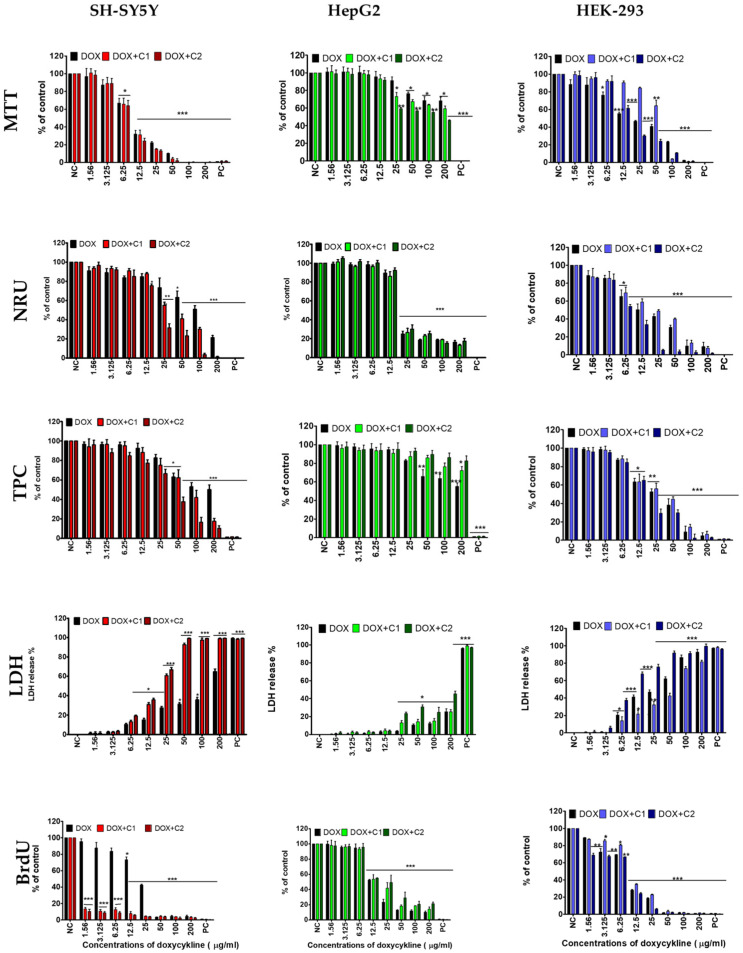
The inhibitory effect of doxycycline (DOX) and its interaction with cannabidiol (C) at concentrations of 1.56 µg/mL (DOX + C1) and 3.12 µg/mL (DOX + C2) on three human SH-SY5Y, HepG2, and HEK-293 cells. The study was performed using five assessment tests: mitochondrial activity (MTT test), lysosomal activity (NRU test), proliferation (TPC test), cell membrane integrity (LDH test), and DNA synthesis (BrdU test). Data are presented as mean ± SD (standard deviation) (*n* = 3). Negative control (NC); positive control (PC). (* *p* ≤ 0.05, ** *p* ≤ 0.01, *** *p* ≤ 0.001).

Doxycycline inhibited mitochondrial action in SH-SY5Y cells, starting at a concentration of 6.25 µg/mL (*p* ≤ 0.05). The drug at 12.5 µg/mL inhibited DNA synthesis and induced cell membrane breakdown. At 50 µg/mL, a decrease in cell proliferation and lysosomal activity was observed (Figure 1).

The presence of CBD in the doxycycline mixtures reduced the drug concentrations required to elicit effects on most of the endpoints studied. Exposure of neuronal cells to both mixtures resulted in inhibition of DNA synthesis at a lower drug concentration (1.56 µg/mL) compared to doxycycline alone (50 µg/mL). Cell membrane disruption, as well as inhibition of lysosomal activity and cell proliferation, occurred at two-fold lower drug concentrations in the DOX + C2 mixture (which contained a higher concentration of CBD)—6.25 µg/mL and 25 µg/mL, respectively—compared to the DOX + C1 mixture and doxycycline alone (Figure 1). No effect of either DOX + C mixture on mitochondrial activity was observed in SH-SY5Y cells compared to doxycycline alone.

**Figure 2:**
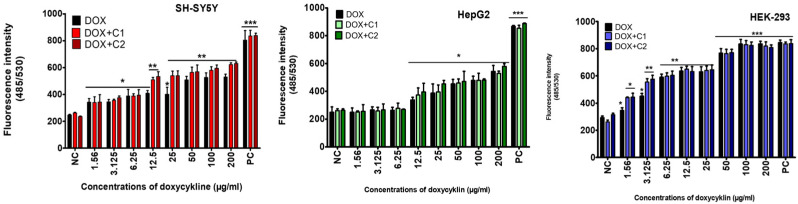
Production of reactive oxygen species (ROS) after 72 h of treatment of SH-SY5Y, HepG2, and HEK-293 cells with doxycycline and co-treatment with CBD at concentrations of 1.56 µg/mL (DOX + C1) and 3.12 µg/mL (DOX + C2). Data are presented as mean ± SD (standard deviation) (*n* = 3) (* *p* ≤ 0.05; ** *p* ≤ 0.01; *** *p* ≤ 0.001).

An increase in free radical production in cultured neuronal cells (SH-SY5Y), accompanied by cytotoxicity, was observed starting from the lowest doxycycline concentration (1.56 µg/mL) also in both tested mixtures with CBD (Figure 2).

The IC_50_ values for doxycycline and its two mixtures with CBD (DOX + C1 and DOX + C2) in SH-SY5Y neuronal cells revealed that the lowest IC_50_ values were observed in the MTT and BrdU assays. In all assays, IC_50_ values decreased with increasing CBD concentration in the mixtures. In the BrdU assay, a significant reduction in IC_50_ values were observed for both mixtures, falling below the lowest tested concentration (1.56 µg/mL), compared to doxycycline alone (Table 1).

A decrease in HepG2 cell viability was observed after exposure to higher doxycycline concentrations compared to SH-SY5Y neuronal cells. The drug inhibited DNA synthesis in HepG2 cells at 12.5 µg/mL. Increasing the drug concentration to 25 µg/mL inhibited lysosomal activity, while a further increase to 50 µg/mL impaired mitochondrial function, disrupted membrane integrity, and reduced cell proliferation (Figure 1).

A low concentration of CBD in the DOX mixture (DOX + C1) increased the drug concentration required to inhibit HepG2 cell proliferation to 200 µg/mL (Figure 1). In contrast, the presence of CBD reduced the DOX concentration required to induce mitochondrial dysfunction and cell membrane damage to 25 µg/mL—half the concentration needed to achieve the same effect with DOX alone. No significant effect of CBD in combination with DOX on DNA synthesis or lysosomal activity was observed in HepG2 cells compared to treatment with doxycycline alone.

It should be noted that a significant increase in free radical production was observed in cultured HepG2 cells following exposure to doxycycline at 12.5 µg/mL, as well as in the tested mixtures with CBD (Figure 2).

The lowest IC_50_ values for DOX and its two mixtures with CBD (DOX + C1 and DOX + C2) were observed in the NRU assay, compared to the BrdU assay in HepG2 liver cells.

In both methods, an increase in IC_50_ values was observed only for the DOX + C1 mixture. In contrast, a decrease in IC_50_ values was noted for the DOX + C2 mixture in four assays—MTT, NRU, LDH, and BrdU—compared to the DOX + C1 mixture (Table 1).

The highest sensitivity to DOX was exhibited by kidney cells (HEK-293). The low concentration of the drug (3.12 µg/mL) inhibited DNA synthesis. At a higher concentration of 6.25 µg/mL, it inhibited both mitochondrial and lysosomal activity and caused degradation of the cell membrane. At 12.5 µg/mL, it inhibited kidney cell proliferation (Figure 1).

The presence of CBD in both DOX + C1 and DOX + C2 mixtures increased the doxycycline concentrations required to inhibit mitochondrial activity, reaching 50 µg/mL and 12.5 µg/mL, respectively, compared to doxycycline alone (Figure 1). However, a high concentration of CBD in the DOX + C2 mixture reduced the effective doxycycline concentration needed to inhibit DNA synthesis to 1.56 µg/mL, compared to doxycycline alone and the DOX + C1 mixture. CBD in both tested mixtures did not affect lysosomal activity, cell membrane integrity, or cell proliferation compared to doxycycline alone (Figure 1).

**Figure 3:**
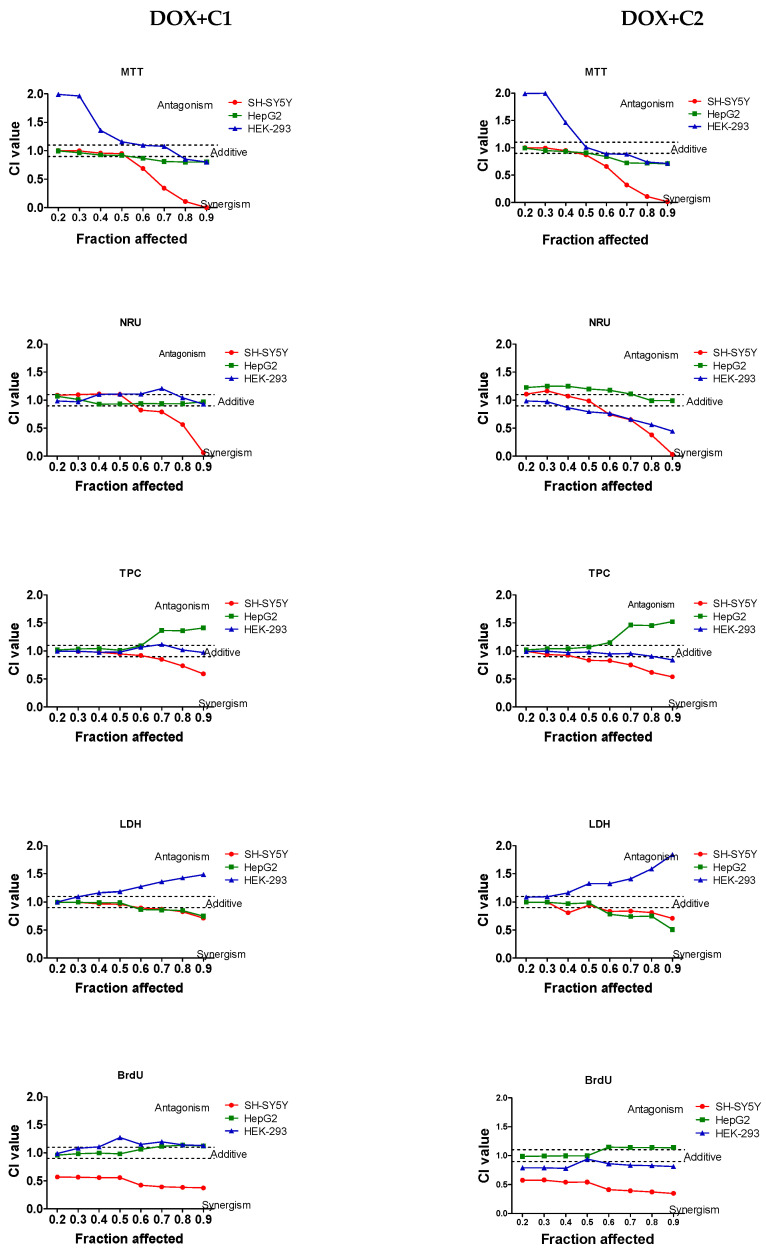
Combination index (CI) and fraction affected (FA) graphs for the combination of DOX and CBD at low concentration (1.56 µg/mL; DOX + C1) and higher concentration (3.12 µg/mL; DOX + C2) in cell cultures of SH-SY5Y, HepG2, and HEK-293 cells using five different assays. Vertical bars represent 95% confidence intervals for CI values based on sequential deletion analysis. Horizontal dashed lines indicate the additive threshold, separating synergistic and antagonistic interactions.

An increase in free radical (ROS) production was observed in cultured kidney cells (HEK-293), starting from the lowest tested concentration of doxycycline, as well as in both tested mixtures. The presence of CBD in both mixtures induced stronger ROS production than doxycycline alone at the two lowest concentrations (1.56 and 3.125 µg/mL) (Figure 3).

The lowest IC_50_ values for the drug and its two mixtures with CBD were observed in the BrdU assay compared to the MTT, NRU, TPC, and LDH assays in HEK-293 kidney cells. A statistically significant increase in the IC_50_ values was observed for the DOX + C1 mixture in the MTT, TPC, and LDH assays. In contrast, a decrease in IC_50_ values was observed for the DOX + C2 mixture in the same assays (Table 1).

### 2.2. The Type of Interaction Between Cannabidiol and Doxycycline

The nature of the interaction between the drug and CBD depended on the cell type as well as the CBD and doxycycline concentration in each mixture. The CI values for the DOX + CBD mixtures are presented in Table 1. Figure 2 shows the CI values calculated across a wide range of cytotoxicity levels (from 20% to 90%) for both combinations. 

The decrease in IC_50_ values for both mixtures compared to the drug alone indicated a synergistic interaction in the MTT and LDH assays in SH-SY5Y cells (Table 1). Strong synergistic effects were observed in neuronal cells at high drug concentrations, whereas additive effects predominated at low doxycycline concentrations in both mixtures across the MTT, NRU, TPC, and LDH assays (Figure 3). Regardless of the drug concentrations, a strong synergistic effect was observed in the BrdU assay for both mixtures (Figure 3).

The increase in IC_50_ values for both mixtures suggests additive and antagonistic interactions in the NRU and BrdU assays, respectively, for HepG2 cells (Table 1). Regardless of the drug concentration, the DOX + C1 mixture exhibited an additive interaction in NRU and BrdU assays (Figure 3). Synergistic effects were observed in HepG2 cells at high drug concentrations, whereas additive effects predominated at low doxycycline concentrations in DOX + C1 mixture across the MTT and LDH assays. In TPC assay, additive and antagonistic effects were shown at low and higher concentrations, respectively. In contrast, the interaction pattern of the DOX + C2 mixture varied with drug concentration. At low DOX concentrations, an antagonistic effect was observed in the NRU assay and an additive effect in the MTT, LDH, TPC and BrdU assay. At higher concentrations of drug, the antagonistic (protective) effect was particularly evident in the BrdU and TPC assays (Figure 2). This protective effect is supported by a rise in IC_50_ value in the BrdU assay and the shift in the DOX concentration in the TPC assay, indicating the influence of CBD on drug cytotoxicity. In the MTT and LDH assays, an additive effect was observed for both mixtures at low drug concentrations, whereas at higher concentrations, a synergistic effect was noted when DOX was combined with CBD.

In HEK-293 cells, the nature of the interaction depended on the concentration of CBD in the mixture. Antagonistic effects were observed at low drug concentrations, whereas additive effects predominated at higher doxycycline concentrations in both mixtures across the MTT. In the NRU assay, additive effects were observed in the DOX + C1 mixture, whereas in the DOX + C2 mixture, additive effects predominated at low drug concentrations, but synergistic effects prevailed at higher doxycycline concentrations. In TPB, an additive effect was observed for both mixtures. In LDH assay, additive and antagonistic effects were shown at low and higher concentrations, respectively, in both mixtures. The DOX + C1 mixture exhibited an additive effect in the BrdU assays. An antagonistic effect was observed in the remaining assays in the DOX + C2 mixture (Table 1, Figure 3).

### 2.3. The Morphological Changes and Death of Cells

Exposure to DOX and its two mixtures with CBD induced morphological changes in SH-SY5Y, HepG2, and HEK-293 cells (Table 2). Drug treatment resulted in cell aggregation followed by detachment from the adhesive surface. The DOX + CBD mixtures caused cell shrinkage, leaving only thin cytoplasmic connections between adjacent cells. Additionally, cell rounding and separation were observed (Table 2).

Exposure of SH-SY5Y cells to DOX increased the proportion of apoptotic and necrotic cells. In contrast, treatment with DOX–CBD mixtures reduced both populations compared to the drug alone. This effect coincided with a decrease in the total cell number, suggesting a synergistic cytotoxic interaction (Table 2). Similarly, in HepG2 cells, doxycycline induced apoptosis and necrosis, whereas both DOX + C mixtures reduced the proportion of apoptotic cells relative to doxycycline treatment alone (Table 2). These findings indicate that CBD may attenuate DOX-induced apoptosis in HepG2 cells. In HEK-293 cells, doxycycline and the DOX + C1 mixture increased apoptotic and necrotic cell fractions, while the DOX + C2 mixture slightly reduced both parameters (Table 2).

## 3. Discussion

### 3.1. Cytotoxicity of Doxycycline

Monitoring studies have shown that doxycycline is one of the most frequently detected antibiotics in both food and the environment. Although it is widely used in medicine, exposure to low concentrations of this drug promotes the development of antibiotic resistance and contributes to adverse effects in humans. Studies conducted on various human cell lines have demonstrated that extended incubation (96 h) with doxycycline, at concentrations ranging from 0.1 to 10 µg/mL, leads to cytotoxic effects through increased lactate production and decreased oxygen consumption. A concentration of 1 µg/mL has been observed to inhibit proliferation and induce apoptotic cell death in the tested cell cultures [43]. In our studies, we observed these effects after only 72 h of exposure in human cell cultures.

Doxycycline exhibits significant pharmacokinetic properties, including the ability to penetrate all organs and tissues, allowing it to reach therapeutic concentrations even in the central nervous system [44]. Neurotoxic effects of doxycycline have been observed during sclerotherapy procedures [44,45,46,47]. Based on available studies, the neurotoxicity of this drug depends on both the duration of exposure and the type of neuronal cells involved. For instance, studies using the rat neuronal cell line PC12 revealed no signs of neurotoxicity after 24 h of exposure to doxycycline at a concentration of 20 µM (10.26 µg/mL) [48]. Interestingly, in SH-SY5Y human neuroblastoma cells, doxycycline at concentrations ranging from 4 ng/mL to 4.4 µg/mL demonstrated a protective effect after 24 h of exposure by reducing the production of reactive oxygen species (ROS) [49]. However, another study reported that a 24 h incubation with doxycycline at higher concentrations up to 4 µg/mL decreased SH-SY5Y cell density—likely indicating a toxic effect—although the effect was minimal at 1 µg/mL [50]. Following a 48 h exposure, the IC_50_ value for doxycycline in SH-SY5Y cells was determined to be 3.7 ± 0.1 µM (1.9 µg/mL) [51]. Additionally, some studies have reported that doxycycline exhibits antitumor activity in neuronal cells [51,52]. In our study, 72 h exposure of SH-SY5Y neuronal cells to 6.25 µg/mL doxycycline decreased cell viability, mainly due to the inhibition of mitochondrial activity. The calculated IC_50_ value for doxycycline, depending on the cellular parameter analyzed, ranged from 9.8 to >200 µg/mL. An increase in free radical production, accompanying cytotoxicity, was observed even at the lowest tested concentration (1.56 ug/mL). Moreover, exposure to doxycycline was associated with a marked increase in apoptotic and necrotic cell populations in the culture. Clear morphological changes in SH-SY5Y cells were observed after exposure to the drug.

Among the numerous contraindications and adverse effects associated with doxycycline use, liver and kidney dysfunction are frequently reported. Studies have indicated a correlation between long-term doxycycline use and the severity of liver dysfunction [53,54,55]. In vitro studies using various hepatoma cell lines have shown that doxycycline can induce several effects indicative of potential hepatotoxicity, primarily related to mitochondrial damage and oxidative stress. Exposure of HepG2 cells to 20 µM (10.3 µg/mL) doxycycline resulted in reduced cell viability due to inhibited mitochondrial activity, cell membrane damage, and increased production of reactive oxygen species (ROS) [56]. Another study demonstrated that doxycycline induces lipid accumulation, mitochondrial membrane depolarization, and a dose-dependent increase in ROS levels, with concentrations of approximately 20–25 µM (10.3–13.0 µg/mL) causing about a 50% reduction in HepG2 cell viability [55,57]. The exposure (72 h) also resulted in mitochondrial inhibition, with an IC_50_ of 20 µM (10.3 µg/mL) [58]. In contrast, studies using human pluripotent stem cell-derived hepatocytes showed that 32 µM (16.5 µg/mL) doxycycline depolarized the mitochondrial membrane and caused significant lipid accumulation, although ROS levels were not significantly elevated [58,59]. Our results are consistent with the studies cited above. We observed that exposure to doxycycline at a concentration of 12.5 µg/mL primarily inhibited DNA synthesis in HepG2 cells, accompanied by increased ROS production. The calculated IC_50_ values, depending on the cellular parameter evaluated, ranged from 13.4 to >200 µg/mL. As in neuronal cells, exposure to doxycycline led to an increase in the number of apoptotic and necrotic cells, along with visible changes in HepG2 cell morphology.

Another adverse effect of DOX is renal impairment. Recent clinical case reports have indicated the occurrence of acute interstitial nephritis following doxycycline administration. The drug’s nephrotoxicity is likely related to its tendency to accumulate in renal tissue [60,61]. In vitro studies have shown that doxycycline at a concentration of 20 µg/mL reduces the viability of HEK-293 cells, although it does not induce apoptosis. The IC_50_ value for doxycycline was reported to be 11.85 µM (6.05 µg/mL) after 48 h of exposure [51]. Additional studies demonstrated that doxycycline at 20 µg/mL also inhibited DNA synthesis in normal HK-2 cells without triggering apoptosis. However, cells of neoplastic origin were more sensitive to the drug compared to normal cells. Doxycycline significantly inhibited protein synthesis and mitochondrial complex activity at a concentration of 5 µg/mL, while a concentration of 10 µg/mL induced apoptosis in renal cancer cells [62]. Doxycycline exerts mitochondrial effects due to the structural similarity between bacterial and mitochondrial ribosomes. Furthermore, it inhibits the activity of matrix metalloproteinases (MMPs), which have a significant role in cancer progression, including renal cell carcinoma [63]. However, the precise mechanism underlying doxycycline’s cytotoxic effects on renal cancer cells remains unclear. Our study demonstrated that 72 h exposure of normal HEK-293 cells to 3.125 µg/mL doxycycline resulted in nephrotoxic effects, primarily through inhibition of DNA synthesis. Cytotoxic effects were accompanied by increased ROS production, even at the lowest tested concentration. The calculated IC_50_ values for doxycycline, depending on the cellular parameter assessed, ranged from 8.9 to 30.4 µg/mL. In contrast to previous reports, we observed a significant increase in apoptotic and necrotic cells in culture following exposure. These cytotoxic effects were also associated with noticeable morphological changes in HEK-293 cells. Our research shows that DOX can cause undesirable effects in humans at the cellular level. Therefore, the challenge was to minimize this effect of the drug. Doxycycline mustn’t cause harmful interactions with other drugs or substances administered concurrently [64].

### 3.2. Effect of Cannabidiol on the Drug’s Cytotoxicity

The ethnological, economic, and well-documented therapeutic relevance of *Cannabis sativa* has driven growing scientific interest in its bioactive compounds. 

In the present study, cannabidiol (CBD) exhibited a synergistic effect in SH-SY5Y neuroblastoma cells, enhancing the cytotoxicity of doxycycline (DOX). Our findings further indicate that the combined DOX + CBD treatment exacerbates mitochondrial dysfunction in SH-SY5Y cells, leading to elevated ROS generation and subsequent cell death. Both compounds are known to disrupt the mitochondrial membrane potential (MMP), promoting cytochrome *c* release and activation of the caspase cascade (caspase-9 → caspase-3) in cancer cells. Although a detailed mechanistic analysis was not performed, pronounced DNA fragmentation was observed following DOX + CBD exposure, as revealed by BrdU incorporation and Hoechst 33342 staining, suggesting caspase-3–dependent apoptosis. Future studies should explore the potential involvement of apoptosis-inducing factor (AIF) translocation and PARP cleavage, both of which are indicative of caspase-mediated cell death. While Hoechst/PI staining supported apoptosis-related changes, definitive discrimination between apoptosis and necrosis requires further validation using Annexin V/PI assays and the detection of cleaved PARP and caspase-3. These analyses will be incorporated into future experiments to confirm and extend the present findings. Numerous reports have confirmed the synergistic potential of CBD in cancer therapy [65,66,67,68,69,70,71,72]. In paclitaxel-based chemotherapy, CBD has demonstrated dual functionality—alleviating pain while enhancing the drug’s therapeutic efficacy [66]. A similar synergistic antiproliferative effect has been observed with CBD in combination with anticancer drugs such as mitoxantrone or cisplatin in melanoma cells [67]. Moreover, studies in canine urothelial cancer have shown that CBD combined with mitoxantrone or vinblastine further enhances the antitumor response [68]. During anticancer therapy, the administration of CBD has been reported to protect healthy cells and improve their function [69,70,71].

In the present study, an antagonistic interaction between DOX and CBD was observed in hepatic (HepG2) and renal (HEK-293) cell cultures, as evidenced by increased IC_50_ values for DOX + CBD mixtures. Notably, this protective (antagonistic) effect was observed at low DOX concentrations, whereas higher DOX levels induced a shift toward synergism. These findings suggest that CBD mitigates DOX-induced cytotoxicity in normal cells, reducing organelle damage and apoptosis, while potentiating DOX activity in neuronal tumor cells. The IC_50_ was dependent on CBD concentration—higher CBD levels were associated with a diminished protective effect. CBD effectively preserved cellular organelle integrity without altering free radical production and displayed anti-apoptotic activity in liver and kidney cell cultures. Previous studies have demonstrated the anti-apoptotic and cytoprotective properties of cannabidiol (CBD), primarily attributed to its antioxidant and anti-inflammatory activities [38,40,73,74,75,76,77,78,79,80]. CBD regulates Nrf2 signaling and enhances the activity of key antioxidant enzymes, including superoxide dismutase (SOD), catalase (CAT), and glutathione peroxidase, thereby reducing oxidative stress and ROS/RNS generation [75,76]. In addition, CBD downregulates pro-inflammatory cytokines such as IL-6 and IFN-α and inhibits the expression of matrix metalloproteinases (MMPs), contributing to reduced cellular inflammation [77]. It also helps maintain ionic homeostasis under stress conditions by modulating Na^+^/Ca^2+^ exchange and promoting calcium storage. The compound supports cell survival, partly through the upregulation of heme oxygenase-1 (HO-1) [78]. Unlike other cannabinoids, CBD exhibits low affinity for classical cannabinoid receptors [78], yet it activates transient receptor potential (TRP) channels, including TRPA1, TRPV1, and TRPV4, even at nanomolar concentrations, which further enhances its cytoprotective effects. The multifaceted effects of CBD are well documented in the literature [65,66,67,68,69,70,71,72,73,74,75,76,77,78,79,80,81]. This compound has demonstrated therapeutic potential in various models of toxic organ injury (e.g., CCl_4_, cadmium, alcohol), where it reduced oxidative stress, inhibited inflammatory mediators, and decreased apoptosis [35,36,72,73,74,75,76,77,78,79,80]. In alcohol-induced liver injury, CBD reduced lipid accumulation and oxidative stress, stimulated autophagy, and modulated the inflammatory response. Its administration has also been shown to inhibit apoptosis and promote cell survival [69,78]. When considering interactions between CBD and doxycycline (DOX), the involvement of cytochrome P450 should also be taken into account. Literature data indicate that both compounds are metabolized by different cytochrome P450 isoforms. Regarding cytochrome P450 enzymes, CBD has been reported to be a potent inducer of CYP1A1 expression [81], whereas doxycycline acts as an inducer of CYP3A4 [4,43,44]. Therefore, most interactions involving doxycycline are attributed to its chelating and physicochemical properties rather than to cytochrome P450–mediated metabolism [44].

## 4. Materials and Methods

### 4.1. Materials

Analytical standard of doxycycline hyclate (CAS: 24390-14-5; m.w. 512.9 g/mol nr cat. D9891) was purchased from Sigma-Aldrich, Poznan, Poland. Cannabidiol (CAS: 13956-29-1; m.w. 314.5 g/mol nr cat. 85705) was obtained from PhytoLab GmbH & Co. KG (Vestenbergsgreuth, Germany). Others chemicals used to cell cultures or made tests: triton X-100, hydrogen peroxide, trypan blue, dimethyl sulfoxide (DMSO), fetal bovine serum (FBS), neutral red dye (NR), Coomassie brilliant blue R-250 dye, 3-(4,5-dimethylthiazol-2-yl)-2,5-diphenyltetrazolium bromide (MTT), Hoechst 33342, propidium iodide, DCFH-DA, hydrogen peroxide, trypsin-ethylenedinitrilotetraacetic acid (EDTA) and antibiotic solution were purchased from Sigma–Aldrich Co. (Poznan, Poland). All other chemicals were purchased from commercial suppliers and were of the highest available purity.

#### 4.1.1. Cell Line and Culture Conditions

The culture conditions of the tested cells were described in detail in our previous publication [38].

#### 4.1.2. Metabolic Activity (MTT Assay)

The metabolic activity of viable cells was assessed by measuring dehydrogenase activity [82]. After exposing the cell cultures to the test compounds, 10 µL of MTT solution (5 mg/mL in PBS) was added to each well of a 96-well plate. After 3 h of incubation, the MTT solution was removed, and the intracellular formazan was dissolved in 100 µL of DMSO. The plate was shaken for 15 min at room temperature and transferred to a Synergy 2 Multi-Mode Microplate Reader (BioTek^®^ Instruments Inc., Winooski, VT, USA) to measure absorbance at 570 nm, using a blank as reference. Cytotoxicity was expressed as a percentage relative to the negative control [83].

#### 4.1.3. Lysosomal Activity (NRU Assay)

This method is based on staining viable cells with neutral red, a dye that readily diffuses through the plasma membrane and accumulates in lysosomes [84]. After incubation with the test compounds, the medium containing the substances was removed, and the cells were washed with PBS. Subsequently, 100 µL/well of neutral red (NR) solution (50 µg/mL) was added and incubated for 3 h. After incubation, the cells were washed again with PBS. The dye retained in viable cells was extracted using a solution of acetic acid, ethanol, and water (1:50:49, *v*/*v*/*v*). After 10 min of shaking, the absorbance of the extracted dye was measured at 540 nm using a Synergy 2 Multi-Mode Microplate Reader (BioTek^®^ Instruments Inc., Winooski, VT, USA), with a blank as reference. Cytotoxicity was expressed as a percentage relative to the negative control [83].

#### 4.1.4. Total Cellular Protein (TPC Assay)

This assay is based on staining total cellular proteins as a measure of proliferation [85]. After incubation, the medium containing the test compounds was removed, and 100 µL of Coomassie Brilliant Blue R-250 dye was added to each well. The plate was shaken for 10 min. The dye was then removed, and the cells were rinsed twice with 100 µL of washing solution (glacial acetic acid/ethanol/water, 5:10:85, *v*/*v*/*v*). Next, 100 µL of desorbing solution (1 M potassium acetate) was added, and the plate was shaken again for 10 min. Absorbance was measured at 595 nm using the Synergy 2 Multi-Mode Microplate Reader (BioTek^®^ Instruments Inc., Winooski, VT, USA), with a blank as reference. Cytotoxicity was expressed as a percentage relative to the negative control [83].

#### 4.1.5. Integrity of Cellular Membrane (LDH Leakage Assay)

Cell membrane integrity was assessed by measuring lactate dehydrogenase (LDH) release [86], using a commercially available Cytotoxicity Detection Kit (LDH) (Roche Diagnostics, Warsaw, Poland). After incubation, 100 µL of culture medium (cell-free) from each well was transferred to the corresponding wells of an optically clear 96-well flat-bottom microplate, and 100 µL of reaction mixture was added to each well. The plate was then incubated for 30 min at room temperature in the dark. To stop the reaction, 50 µL of 1 M HCl was added to each well. Absorbance was measured at 492 nm using the Synergy 2 Multi-Mode Microplate Reader (BioTek^®^ Instruments Inc., Winooski, VT, USA), with a blank as reference [83].

#### 4.1.6. DNA Synthesis (BrdU Assay)

Cell proliferation was evaluated using the BrdU assay (Cell Proliferation ELISA, BrdU [colorimetric], Roche, Switzerland) according to the manufacturer’s instructions. Briefly, BrdU was incorporated into newly synthesized DNA in place of thymidine during cell division. Incorporated BrdU was detected using an anti-BrdU antibody conjugated with peroxidase. The enzymatic reaction product was quantified by measuring absorbance. Absorbance was measured at 370 nm and 492 nm using the Synergy 2 Multi-Mode Microplate Reader (BioTek^®^ Instruments Inc., Winooski, VT, USA), with a blank as reference.

#### 4.1.7. Oxidative Stress (DCFH Assay)

Intracellular ROS levels were evaluated using the redox-sensitive fluorescent dye DCFH-DA [87]. Briefly, the cells were incubated with 5 μM DCFH-DA for 1 h at 37 °C in the dark. Thereafter, the tested compounds were added at the concentrations used in the study. Cells treated with 500 μM hydrogen peroxide (H_2_O_2_) served as a positive control. After 72 h of incubation, the fluorescence of 2′,7′-dichlorofluorescein (DCF) was measured. Fluorescence was recorded at 485/530 nm using a fluorescence microplate reader (BioTek^®^ Instruments Inc., Winooski, VT, USA).

#### 4.1.8. Apoptosis and Necrosis Death Cells

The type and extent of cell death in the control and treated cultures were evaluated using fluorochrome staining with Hoechst 33342 and propidium iodide to assess apoptosis and necrosis [88]. Dead cells were analyzed morphologically using a fluorescence microscope (Axiovert 200 M, Zeiss, Oberkochen, Germany). Cells with pink, fluorescent nuclei were considered necrotic, whereas cells with blue, fluorescent nuclei (fragmented and/or condensed chromatin) were classified as apoptotic.

#### 4.1.9. Cellular Morphology Analysis, (May–Grünwald–Giemsa (MGG) Staining)

This method was used to illustrate changes in cell morphology under the influence of the tested compounds. The staining was performed on Lab-Tek plates (Nunc, NY, USA). Cells at a density of 1 × 10^5^ cells/mL were used. After 72 h of incubation with or without the tested drugs (control), the culture medium was removed, and the cells were stained with 1 mL of May–Grünwald dye for 3 min at room temperature. Then, 1 mL of deionized water was added to each plate. After another 3 min of incubation at room temperature, the liquid was removed. All plates were rinsed with deionized water, and the cells were subsequently stained with Giemsa dye (dilution 1:20) for 30 min at room temperature. After staining, the dye was removed, the wells were rinsed with 1 mL of deionized water, and the plates were allowed to dry. Images were captured using a light microscope (Zeiss, Oberkochen, Germany).

#### 4.1.10. Assessment of Synergistic/Antagonistic Effects

To determine the types of interaction that occur when cells are exposed to combinations of DOX and CBD, the method established by Chou and Talalay [89] was used in this study. The dose–effect relationships of the individual and combined test compounds were modeled biometrically using the Median-Effect Equation based on the Mass Action Law.

fa/fu = (D/Dm)^m^

D—dose of the drug or CBD; fa—fraction affected by D; fu—fraction unaffected (i.e., fu = 1 − fa); Dm- median-effect dose (e.g., IC50); m—coefficient signifying the shape of the dose–effect relationship (m = 1, m > and m < 1 indicated hyperbolic, sigmoidal, and flat sigmoidal dose–effect curves, respectively).

In this isobolographic analysis, the combination index (CI) is a quantitative parameter used to evaluate the type and strength of interactions between multiple compounds. For all combinations, CI values were calculated across a range of affected fractions (Fa) from 0.05 to 0.95 (corresponding to 5–95% toxicity). CI values of 0.9–1.1, <0.9, and >1.1 indicate additive, synergistic, and antagonistic effects, respectively.

#### 4.1.11. Analysis of Statistical Data

The study was conducted in three independent experiments (*n* = 3). The results are presented as mean values ± standard deviation (SD). Cytotoxicity data were analyzed using one-way analysis of variance (ANOVA), followed by Dunnett’s post hoc test to assess significance relative to the negative control. The half-maximal inhibitory concentration (IC_50_), representing the drug or drug–CBD mixture concentration required to inhibit cell viability by 50%, was calculated using GraphPad Prism 5.0. Statistical comparisons between IC_50_ values were performed using ANOVA followed by Tukey’s post hoc test. Differences were considered statistically significant at *p* ≤ 0.05.

## 5. Conclusions

To summarize our research findings, we conclude that the interactions between doxycycline and cannabidiol (CBD) are cell type-dependent. In neuronal cancer cells, the simultaneous use of both compounds produced a synergistic effect, enhancing anticancer activity. In contrast, in liver and kidney cells, the protective effects depended on the concentrations of both CBD and doxycycline. These results suggest that cannabidiol may be a promising candidate for preventing doxycycline-induced damage and dysfunction in healthy cells. However, further research is needed, including studies involving other cell types, time-course experiments, analysis of additional protective mechanisms of CBD, and preclinical studies in animal models. It should also be noted that the growing use of CBD in medicine, veterinary medicine, and public health underscores the need for further investigation. The effects of veterinary drug residues in food and the environment on human cells should be clarified using various cell models. Particular attention should be given to CBD’s interactions and potential protective effects, as its interactions with drug residues in food remain unexplored. Further research is required to assess the effects of CBD during long-term dietary exposure.

## Figures and Tables

**Table 1:** The values of IC_50_ (µg/mL) for doxycycline (DOX) and its mixtures with CBD ((DOX + C1) and (DOX + C2)) calculated in SH-SY5Y, HepG2, and HEK-293 cells using the MTT, NRU, TPC, LDH, and BrdU assays after 72 h exposure. Data are presented as mean ± SD (standard deviation) (*n* = 3).

Cell Lines	Assay	DOX	DOX + C1	CI	DOX + C2	CI
SH-SY5Y	MTT	9.8 ± 0.9 ^a^	9.4 ± 1.1 ^a^	0.87	10.4 ± 0.4 ^a^	0.85
	NRU	>200	41.0 ± 2.3 ^a^	-	17.7 ± 0.9 ^b^	-
	TPC	>200	69.6 ± 4.3 ^a^	-	35.5 ± 1.4 ^b^	-
	LDH	149 ± 6.5 ^a^	37.6 ± 0.6 ^b^	0.47	18.3 ± 4.3 ^c^	0.09
	BrdU	15.6 ± 0.7 ^a^	<1.56	-	<1.56	-
HepG2	MTT	>200	>200	-	147.6 ± 3.9 ^a^	
	NRU	13.4 ± 0.9 ^a^	17.4 ± 0.9 ^a^	0.94	16.5 ± 0.8 ^a^	0.93
	TPC	>200	>200	-	>200	-
	LDH	>200	>200	-	116.4 ± 4.7 ^a^	-
	BrdU	24.0 ± 0.4 ^a^	43.5 ± 1.9 ^b^	1.97	35.5 ± 1.9 ^c^	1.43
HEK 293	MTT	17.4 ± 1.0 ^a^	88.7 ± 2.1 ^b^	4.35	20.5 ± 0.9 ^a^	0.97
	NRU	12.7 ± 1.0 ^a^	13.6 ± 1.3 ^a^	0.93	11.2 ± 0.9 ^a^	0.90
	TPC	14.8 ± 1.3 ^a^	36.9 ± 2.0 ^b^	2.77	19.7 ± 1.1 ^c^	0.95
	LDH	30.4 ± 2.7 ^a^	62.1 ± 3.8 ^b^	2.09	8.9 ± 0.7 ^c^	0.35
	BrdU	8.9 ± 0.1 ^a^	9.1 ± 0.7 ^a^	0.91	9.3 ± 0.4 ^a^	0.95

The different small letters (a–c) in the lines indicate significant differences among treatment methods (*p* ≤ 0.05). Combination index (CI): a numerical means of the degree of interaction between drugs, indicating an additive (CI = 1), synergistic (CI < 1), or antagonistic (CI > 1) effect for a given endpoint.

**Table 2:** Morphological evaluation of SH-SY5Y, HepG2, and HEK-293 cells (control vs. treatments: DOX (doxycycline); DOX + C1 (doxycycline with cannabidiol at 1.56 µg/mL); DOX + C2 (doxycycline with cannabidiol at 3.125 µg/mL)) after 72 h of incubation, using May–Grünwald staining (200×). Fluorescence images of Hoechst 33342 and propidium iodide staining for each treatment group. Column graphs show the median fluorescence intensity.

	Control	Doxycycline (DOX)	DOX + C1	DOX + C2
SH-SY5Y cells				
May–Grunwald staining	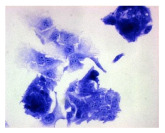	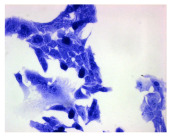	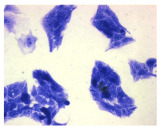	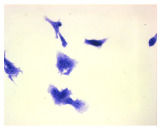
Hoechst 33342 + propidium iodide	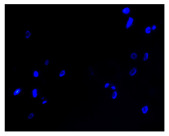	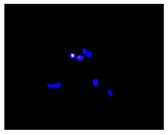	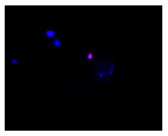	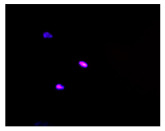
	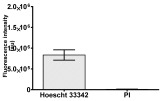	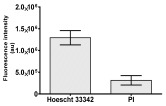	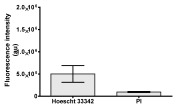	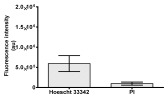
HepG2 cells				
May–Grunwald staining	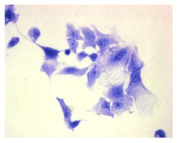	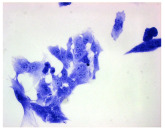	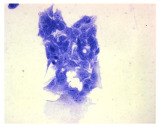	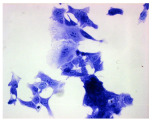
Hoechst 33342 + propidium iodide	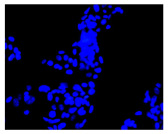	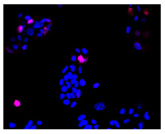	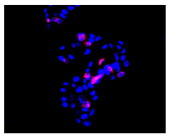	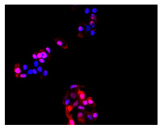
	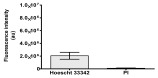	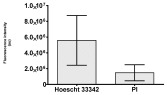	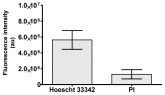	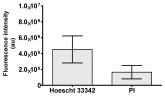
HEK-293 cells				
May–Grunwald staining	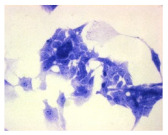	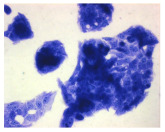	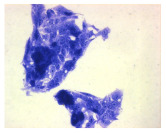	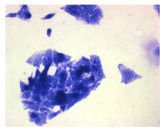
Hoechst 33342 + propidium iodide	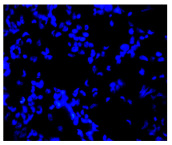	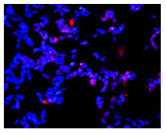	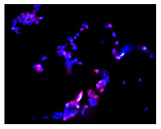	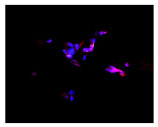
	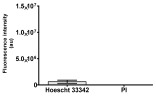	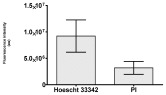	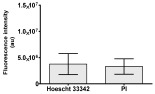	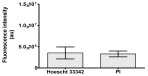

## Data Availability

The original contributions presented in this study are included in the article/Supplementary Materials. Further inquiries can be directed at the corresponding authors.

## Supplementary Materials

The following supporting information can be downloaded at: https://www.mdpi.com/article/10.3390/molecules30214319/s1, Figure S1: The effects of cannabidiol (CBD) on the human SH-SY5Y, HepG2, and HEK-293 cell lines were evaluated using five endpoints: mitochondrial activity (MTT assay), lysosomal activity (NRU assay), proliferation (TPC assay), cell membrane integrity (LDH assay), DNA synthesis (BrdU assay) and oxygen species (ROS) production (DCFH-DA assay).
