# Supplementary figures and images for: The Action of Cannabidiol on Doxycycline Cytotoxicity in Human Cells—In Vitro Study
Source: Molecules. 2025 Nov 6;30(21):4319. doi: 10.3390/molecules30214319 (PMC12610721; doi:10.3390/molecules30214319)

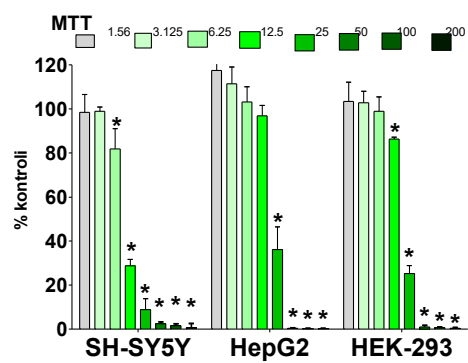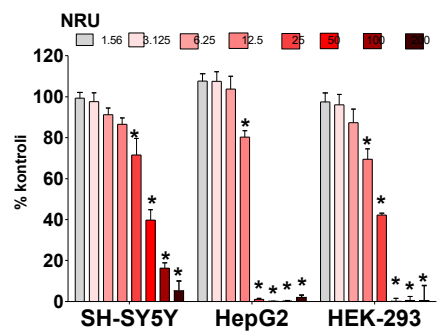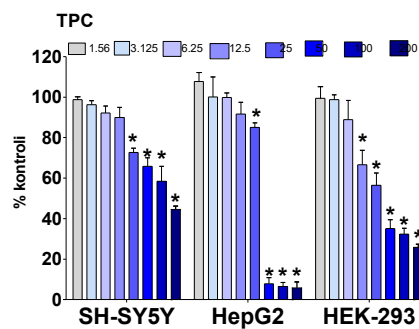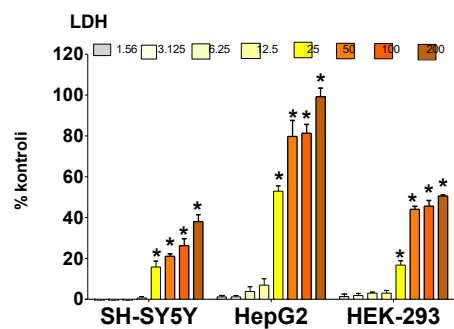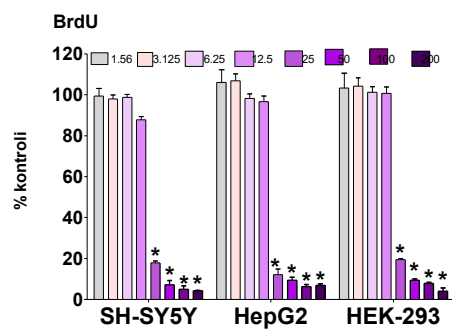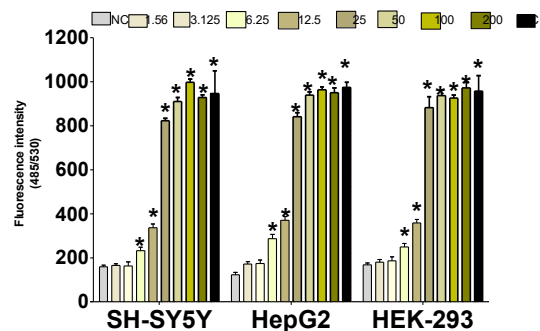

Supplement: Supplementary file 1 [file molecules-30-04319-s001.zip › molecules-3917388-supplementary.pdf]
